# Presence of a Septal Occluder Is No Longer an Obstacle for Pulmonary Vein Isolation by Cryoablation

**DOI:** 10.19102/icrm.2021.120602

**Published:** 2021-06-15

**Authors:** Santiago Giraldo Ramírez, Juan Fernando Agudelo Uribe, Juan David Ramírez Barrera, Rafael Correa Velásquez, Gloria Saenz Jaramillo

**Affiliations:** ^1^Cardiology Fellowship, Universidad de Antioquia, Medellín, Colombia; ^2^Clínica Cardiovid, Medellín, Colombia

**Keywords:** Atrial fibrillation, cryoablation therapy, pulmonary vein isolation, septal occluder device, transseptal puncture

## Abstract

Cryoablation for pulmonary vein isolation in atrial fibrillation has been considered a relative contraindication in the presence of a septal occluder device. We describe the successful conduct of this technique with a multimodality imaging approach.

A 69-year-old man presented to our atrial fibrillation (AF) clinic due to the presence of paroxysmal AF diagnosed five years earlier. He complained of persistent palpitations despite the use of metoprolol succinate (100 mg once daily) and propafenone (150 mg twice daily), and he did not tolerate higher doses of antiarrhythmics due to gastrointestinal complaints. Echocardiography showed a preserved ejection fraction (0.55) and a normal atrial size. He was referred for pulmonary vein (PV) isolation with a cryoballoon due to refractory paroxysmal AF. The patient’s medical history included hypothyroidism and patent foramen ovale (PFO) closure for a cryptogenic stroke with a 25-mm Amplatzer® device (Abbott, Chicago, IL, USA) 15 years earlier. Medications included levothyroxine, apixaban, and statins.

Following the exclusion of left atrial thrombus by transesophageal echocardiography, the patient was taken to the electrophysiology laboratory. Under general anesthesia, femoral vein access was gained. Quadripolar and decapolar catheters (Inquiry®; Abbott) were advanced to monitor His and coronary sinus signals, respectively. The His catheter was used as a reference to mark the most caudal aspect of the aorta and, in this way, to aid in the selection of the transseptal puncture (TSP) site. Intracardiac echocardiography (ICE) (ViewFlex®; Abbott) was used to guide the TSP and to monitor complications during the procedure **(Figure 1)**.

As was described previously by Sang et al.,^1^ to indirectly localize the outflow tract and aortic root (as a white shadow), an inferior cava vein angiography was performed. A fixed-curve sheath (Swartz® Braided LAMP 90®; Abbott) was advanced into the right atrium, and 10 mL of contrast medium was injected with cineangiography film recording in the right anterior oblique (RAO) projection. Opacification of the right atrium, right ventricular outflow tract, and pulmonary artery creates an outline around the aortic root (a white shadow was seen). Thus, this structure can be referenced without the need to inject contrast directly through the aorta.

To enhance the precision and security of the procedure, a quadripolar catheter was positioned in the His area.^2^ The distance between the anterior edge of the Amplatzer® and the proximal pole of the His-located quadripolar catheter was 16 mm. A site between these reference points, inferior to the aortic shadow, was selected as the ideal location for transseptal atrial puncture **(Figure 2)**. An anterior and inferior puncture site was selected to ease cryoballoon manipulation during right PV therapy **(Figure 3)**.

A 0.032-in guidewire and a transseptal introducer sheath (Swartz® Braided LAMP 90®) were advanced. Using a 71-cm transseptal needle (BRK®; Abbott) and under fluoroscopic imaging (in the RAO projection) and direct ICE vision, transseptal catheterization was performed. Afterward, the 0.032-in guidewire was exchanged for a 260-cm-long, high-support hydrophilic-coated guidewire (GLIDEWIRE ADVANTAGE® 0.035 in; Terumo Medical Corporation, Somerset, NJ, USA); then, a FlexCath Advance 15-F® steerable sheath (Medtronic, Minneapolis, MN, USA) was placed in the left atrium **(Figure 2)**.

Guided by ICE and fluoroscopy and taking the computed tomography imaging of the left atrium as an anatomic reference, the cryoballoon and a circular mapping Achieve® catheter (Medtronic) were advanced into the left atrium and subsequently into every PV ostium. Over the Achieve® catheter, the cryoballoon was advanced and insufflated. Iodinated contrast was injected through the distal tip of the catheter to test complete sealing of the PV in order to proceed with the cryoablation therapy for 240 seconds. Continuous phrenic stimulation was performed during right PV cryoablation to detect lesions of the phrenic nerve. At the end of the study, ICE ruled out pericardial effusion. The patient was in sinus rhythm the day after the procedure and was discharged without complications.

## Discussion

A increasing frequency of TSP use has been observed, mainly driven by the acceptance of PV ablation as a strategy for rhythm control in AF. The high success rate of the procedure (> 99%) and the low number of complications (< 1%) make this technique an essential tool for the electrophysiologist.^3^ On the other hand, the presence of a septal occluder device is now a common scenario in clinical practice as its placement is the procedure of choice for the closure of most atrial septal defects and its use has gained adoption for PFO closure since its approval for the management of patients with cryptogenic stroke by the United States Food and Drug Administration in 2016.^4^ Thus, it is not uncommon to find a patient with a septal occluder device and a clinical indication for PV isolation. However, the presence of an atrial septal occluder has classically been considered a relative contraindication for PV isolation.

Radiofrequency catheter ablation was the first technique used for PV isolation in the classic report by Haïssaguerre et al*.*^5^ Since then, the evolution of both techniques and devices has made PV isolation a routine procedure in the area of electrophysiology. Meanwhile, cryoballoon ablation has emerged as an alternative to radiofrequency energy. The equivalent rate of sinus rhythm maintenance at one year in paroxysmal AF (60%–80%)^6,7^ and the shorter time required to complete the procedure have made cryoablation therapy an attractive option.

PV isolation with radiofrequency catheter ablation is a well-described procedure in studies of a few patients with septal occluder devices.^1,8^ In these investigations, TSP had a 100% success rate, and the puncture could be achieved through the native septum in 70% to 90% of cases. In 10% to 30% of patients, the TSP was performed through the device without complications. In our case, due to the presence of paroxysmal AF and the normal atrial size, we chose the cryoballoon ablation technique over radiofrequency ablation, primarily to perform a single TSP.

To the best of our knowledge, there are only two previous reports of TSP for cryoballoon ablation in the presence of a septal occluder device.^9,10^ In both reports, left atrium access was gained through the native septum without the need to cross the device.^9,10^ In the report by Ströker et al.,^9^ TSP was conducted inferoposteriorly to the device, which could make cryoablation therapy for the right PV more difficult to complete due to the very acute angle created to access the right-side veins. Rind et al.^10^ described the use of an anteroinferior puncture site through the native septum without complications.

In our case, the use of ICE was of paramount importance to guide the procedure. Due to the larger sheath required during PV isolation with a cryoablation balloon, the procedure is technically more challenging in the presence of a septal occluder device. In this scenario, it is essential to gain anterior access through the septum as this puncture facilitates advancing the catheter and access into the right PVs.^11^ High anatomic resolution and real-time imaging with the use of ICE allowed us to gain access to the left atrium through the native septum. In this manner, ICE has a central role evaluating the anatomical variants of the interatrial septum, refining the puncture site selection for TSP, and improving the early detection of complications (eg, cardiac tamponade).^12^

One of the advantages of our approach is the use of a quadripolar catheter located in the His signal. Although this is a classic tool to demarcate the superior margin of the interatrial septum (which lies in juxtaposition to the noncoronary cusp of the aortic valve in its most inferior aspect),^2^ this additional point was very helpful because it allowed us to see a reference point during the fluoroscopic view and, hence, avoid an accidental puncture of the aortic root.

## Conclusion

The presence of a septal occluder device in patients with an indication for PV isolation for AF is a situation that is expected to become increasingly common. PV isolation with a cryoablation balloon is a feasible procedure in this scenario. In the present case, the use of multimodal imaging with inferior cava vein angiography, ICE, and fluoroscopy led us to complete the procedure safely.

## Figures and Tables

**Figure 1:**
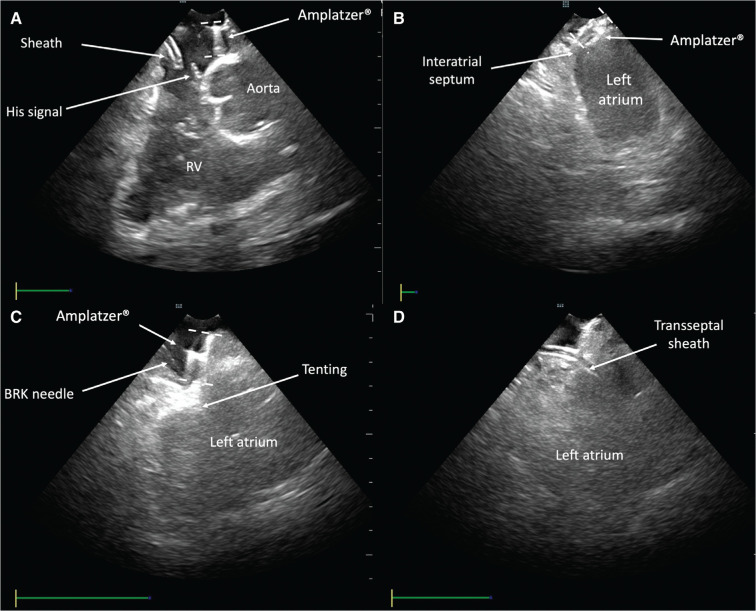
ICE images taken during the procedure. **A:** Home view showing the Amplatzer® device and the His signal catheter. **B:** A point in the native septum was selected for left atrium access. **C:** ICE was used to guide septum puncture. **D:** Access to the left atrium. ICE: intracardiac echocardiography BRK: Brockenbrough; RV: right ventricle.

**Figure 2:**
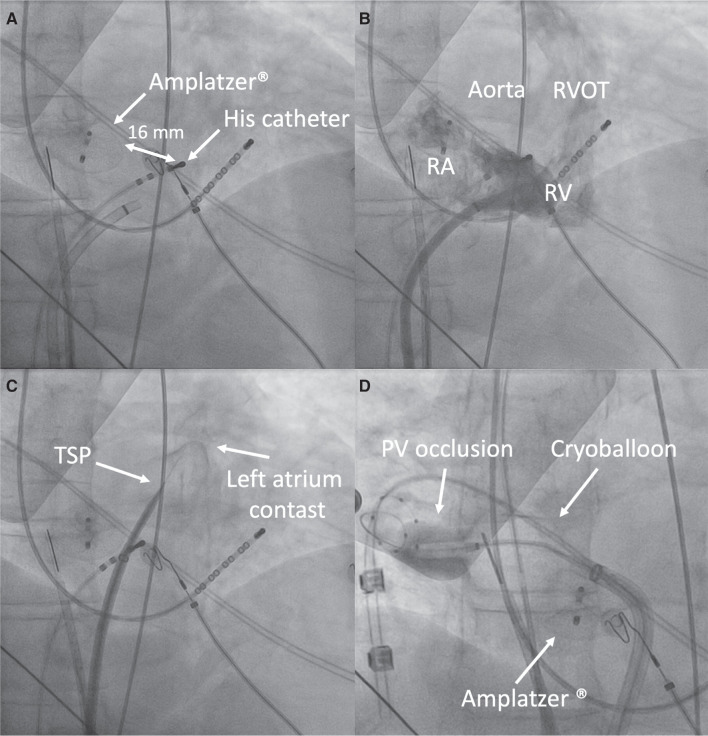
**A:** Distance between the anteroinferior border of the Amplatzer® device and the His signal catheter. **B:** Inferior cava vein angiography. **C:** TSP with successful passage into the left atrium. **D:** Cryoablation therapy. PV: pulmonary vein; RA: right atrium; RV: right ventricle; RVOT: right ventricular outflow tract; TSP: transseptal puncture.

**Figure 3:**
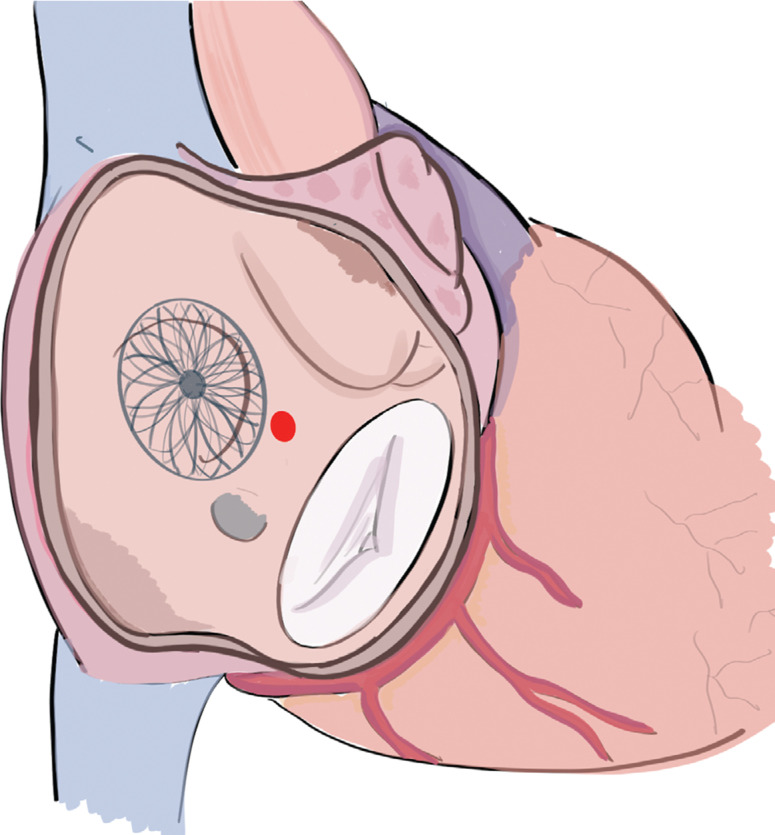
Image showing the anatomical references relevant during the procedure. The Amplatzer® device lies in the interatrial septum. An inferior and anterior point through the native septum (marked as a red point) was chosen for TSP. TSP: transseptal puncture.
